# Book Reviews

**Published:** 1913-11

**Authors:** 


### BOOK REVIEWS

*Books mentioned may be inspected at and ordered through this office. So far as possible, books received in any month will be reviewed in the issue of the second month following.*

---

Digest of Comments on the U. S. Pharmacopoeia. Eighth Decennial Revision, and on the National Formulary, third edition, for the calendar year 1911. By Murray Galt Motter and Martin I. Wilbert. Published by U. S. Public Health Service.

This is a large, paper bound volume of 683 pages. The first 174 pages contain various tables of articles omitted from and added to the Pharmacopœia, fifteen drugs most commonly prescribed, atomic weights, etc., and general comments. The remainder of the work is arranged alphabetically, by official drug titles containing under each criticisms, suggestions as to improvements, modifications of tests, etc. An enormous volume of current medical and even popular literature has passed through the hands of the authors, and they have abstracted and sorted the comments analytically. If any adverse criticism is to be made, it is that they have been too conscientious in including many references of no special critical value. This is, however, a good fault and we trust that the next committee of revision will pay close attention to this work in improving the Pharmacopœia to such a degree that no elaborate argument will be necessary to induce practicing physicians to include it in their libraries.

---

Treatment of Rheumatic Infections, issued by Parke, Davis & Co.; cloth bound; 134 pages.

We understand that this book will be sent gratis to any physician interested. It deals largely with the Schaefer Phylacogens and consists of excerpts from medical literature. In spite of the obvious commercial interest of the publishers, they have shown, as at other times, a commendable impartiality of view. In fact, they have simply reproduced the views of men able to judge impartially. There is no disposition to exaggerate the merits of phylacogen nor to conceal any contraindications; in fact, the contraindications which are, for the most part, obvious, are plainly indicated in the index. Schaefer's theories were carefully tested out by the co-operation of many physicians throughout the country, before the sale of phylacogens was undertaken. Their reports, while showing that not all of the symptomatic conditions commonly called rheumatism were amenable to phylacogen treatment, and that, as was expected from the analogy to syphilis and other infections, residual lesions could not usually be cured, were highly favorable, and this decision has been corroborated by subsequent experience on a scale which eliminates the possibility of personal bias on the part of the physician or of psychic effect on the part of the patient. With due regard to changed conditions, it may fairly be said that the introduction of the phylacogen treatment of rheumatism—using the term in its proper sense—marks an epoch fully as important as the introduction of salicylates many years ago.

---

THE DOCTOR IN COURT, by Edwin Valentine Mitchell, LL.B. of the Massachusetts Bar. The Rebman Co., New York: 152 pages; $1.00.

We like the way in which the author announces himself, barring the personal difference of a sense of allegiance to the city instead of the state and the practical desirability of locating an author, which leads us to suggest that future editions should give his residence in Boston. Imagine a medical man presenting as his credentials for authorship the fact that he was a member of the medical profession, and, therefore, prepared by the experience of his life work to treat the subject in hand.

While this is a brief treatise, it contains a surprising amount of detailed information and even citation of cases. We particularly recommend the reading of the chapters on civil and criminal responsibility and remuneration.

---

THE MICROTOMIST'S VADE MECUM, Arthur Bolles Lee, Baughy sur Clarens, Switzerland. P. Blakiston's Son & Co., Philadelphia; 526 pages; $4.00.

This is the seventh edition of this standard work, the first having appeared in 1885. The scope of the work is broad, including not only the ordinary examinations of normal and pathologic human tissues, but those of the lower animals, with special attention to methods applicable to invertebrates, embryologic study, etc. In the preface, attention is called to the newer methods which have required the bringing out of another edition while the index allows easy reference both by subjects and by names of authors. The processes for preparing tissues for examination are fully discussed, even to methods of killing animalculæ and preserving specimens prior to mounting. While replete with technical details, indicating thorough familiarity with the subject, a simple system of cross references avoids prolixity and repetition and general principles, as of the action of hardening and staining agents, are well discussed.

An Introduction to the Study of Infection and Immunity. Including Serum Therapy, Vaccine Therapy, Chemotherapy and Serum Diagnosis. By Charles E. Simon, M. D., Professor of Clinical Pathology and Experimental Medicine. College of Physicians and Surgeons, Baltimore. New (2d) edition, thoroughly revised. Octavo, 325 pages; illustrated. Cloth, $3.25, net. Lea & Febiger, publishers, Philadelphia and New York, 1913.

In the issue of December, 1912, we reviewed the first edition of this work. The professional appreciation of Dr. Simon's book has necessitated the issue of a second edition, and, at the same time, given opportunity for the inclusion of some new matter along the lines of auto and normal serum therapy, chemo therapy of pneumoccic infection and cancer, serum diagnosis of pregnancy, etc. We have elsewhere expressed the opinion that the method of presentation of the general subject of "Immunology"—a highly objectionable word for which a substitute should be secured before it becomes so firmly established as fibroma and appendicitis—is subject to criticism. This criticism applies no more to this book than to most other writings on the subject and consists in questioning the wisdom of carrying out in graphic detail, plausible but purely hypothetic explanations of the chemic reactions of immunity. It is true that diagrams serve to fix in the mind of the students, the technical terms used in explaining the process of immunity, largely because the terms themselves are metaphors. But is it well to establish a visual image which is probably no nearer the truth than the pictures of germs in Sunday supplements, and is it well to popularize hypothetic explanations which really have no demonstrated basis of literal truth? Is it not more in harmony with the pragmatism of medical science in general to teach observed facts and their practical application and to present hypotheses in the barest form possible?

It should be understood that this criticism applies to the present author only to the extent that he has followed precedent. In his technical descriptions, presentation of statistics of accuracy, and therapeutic and diagnostic advice, the author has ably summarized the state of our present knowledge and, we believe, has anticipated as nearly as is possible, the results of further development.

---

Studies Concerning Glycosuria and Diabetes, Frederick M. Allen, A. B., M. D., Pomona, Cali. Published by W. M. Leonard, Boston; 1179 pages; $9.00.

This work embodies the results of three years' study and research in the laboratory of preventive medicine and hygiene of

Harvard, the author acknowledging a scholarship and other assistance toward the publication, as well as indebtedness for guidance and assistance in the scientific work. The long list of bibliographic references also indicates a disposition to give due credit to authorities consulted. As might be expected from one so candid in acknowledging the part of others, the work is essentially original and of a magnitude—referring not so much to the size of the volume as to the extent of the research implied—which clearly indicates that the author has not only devoted much time and thought to his task, but has borne an expense far beyond the amount of the scholarship, etc.

We assume, from the nature of the research and the time devoted to it in an institution, that the author is a comparatively young man. If so, he has shown a surprising maturity of judgment in weighing clinical evidence along with that derived from laboratory experiment; in following the various theories current, without marked bias; and in avoiding the natural tendency to bring theories and observations together to make a complete, satisfactory, one-sided explanation of the disease in question. The publishers state in their letter of transmittal, that they do not expect a large market for the book, but merely the interest of those who have devoted special study to the subject. We trust that their modest expectations will be exceeded. In spite of the fact that the book emanates from laboratory research and in spite of its magnitude, it is, in our judgment, just the work that the average practitioner of medicine should read, and should preserve for consultation. This is because so many theories are exploded, so many definite facts established and because the author has not "simplified" the subject of diabetes and the more or less related phenomena of glycæmia, glycuria, glycogen formation, etc., at the expense of the truth.

---

Minor and Operative Surgery, Including Bandaging. By Henry R. Wharton, M. D., Professor of Clinical Surgery in the Woman's Medical College, Philadelphia. New (eighth) edition, enlarged and thoroughly revised. 12mo. 700 pages, with 570 illustrations. Cloth, $3.00 net. Lea & Febiger, Philadelphia and New York, 1913.

This book has become a standard, as indicated by the number of editions through which it has passed. The condensed literary style employed, the omission of unnecessary details and the use by the publishers of thin but opaque, non-reflecting, paper, give the impression, at first glance, of a rudimentary treatise. This impression is unjust, for the work is really very comprehensive and thorough, only the rarest conditions and details appropriate to a special monograph or to a general treatise of cyclopædic nature, being omitted. The work is of high order from the didactic standpoint, and the author speaks with the experience of many years as a surgeon and a teacher.

---

A Treatise on the Diseases of Women. For Students and Practitioners. By Palmer Findley, B. S., M. D., Professor of Gynecology, College of Medicine, State University of Nebraska; Gynecologist to the Clarkson Memorial Hospital and Douglas County Hospital; Fellow of the American Gynecolo-ical Society; Fellow of the American Association of Obstetricians and Gynecologists; Fellow of the Chicago Gynecological Society. Octava, 954 pages, illustrated with 632 engravings in the text and 38 plates in colors and monochrome. Cloth, $6.00, net. Lea & Febiger, Philadelphia and New York, 1913.

It gives us great pleasure to review this work of an acquaintance and quondam colleague. Few branches of medicine have produced the volume of literature that has arisen from gynæcology and, while this statement applies particularly to periodical writings, we have been impressed with the idea that books on gynæcology, generally speaking, have attained degrees of magnitude and of elaborateness of treatment, illustration, etc., rather beyond those of other branches. Hence, a new author entering this field has a rather more difficult task than the average, owing to the competitive standards established. While the present work is the outgrowth of "Diagnosis of Diseases of Women," published some years ago, and while Dr. Findley is well known as a gynæcologist, not only in and about Chicago where his earlier work was done, and in the wide field to which Omaha serves as a medical center, but also throughout the gynæcologic world, we need not apologize for speaking of him as a new author, so far as text books of the first magnitude are concerned. A more critical examination of this book shows that its merit does not lie solely in its size, comprehensiveness, nor in the elaborate illustrations. It is not a mere compilation, though, in a work of this kind, careful attention to the work of others than the author is a necessary function of authorship which has not been neglected in the present instance. What impresses us most in Dr. Findley's book is that he has brought to the reader not merely the experience and views of an operator but a careful attention to gynæcology in all its parts, yet without a hint of the old "puttering" office manœuvres. When he speaks of diagnosis, it is not the superficial consideration of history and symptoms and rudiments of palpation and percussion, preliminary to the use of the knife to make an equally superficial inspection of an exposed organ. On the contrary, he manifests personal familiarity with blood examinations, bacteriology and morbid histology, etc., as well as what the late H. F. Formad used so often to emphasize as the preliminary to diagnosis—definite, classified knowledge of what might and what might not be found in any given organ. The same thoroughness is displayed in regard to pre and post-operative treatment and to non-operative methods of therapeutics, including hygiene and dress. Yet, if we were to select any special points for illustrating the value of this book, they would be the definiteness with which pathology and etiology are discussed and the completeness of the descriptions and illustrations of anomalies. And this is only another way of saying that the author has laid his foundations broad and deep.

---

Diseases of Children. Henry Enos Tuley, M. D., Louisville, published by the C. V. Mosby Co., St. Louis, 684 pages, 106 engravings and three colored plates, second revised edition, cloth.

This book discusses, systematically, the peculiarities of infants and children, anatomic, physiologic, dietetic and therapeutics, pays great attention to details of nourishment, especially when it is necessary to rear the child artificially by milk, and then treats of the various diseases, according to their logical classification by specialties. The various formulæ for feeding and the methods of diagnosis by examination of fæces, urine, etc., are described in practical terms, well adapted to routine clinical use. We note some interesting points taken at random: Koplik's spots are reproduced by courtesy of Dr. John Zahorski, in color. This is believed by the publishers to be the first accurate reproduction of the lesion ever published. (By the way, without intending any discourtesy to Dr. Koplik, it should be remembered that the fact of characteristic lesions of the exanthemata occurring on mucous membranes has been taught as a routine by many lecturers, for many years, if not centuries and the same lesions have been clinically demonstrated in cognate courses for students. Some of these students have regularly used these lesions as diagnostic features in practice.) The convenient thermos bottle is mentioned as a possible cause of serious gastro-enteric disturbance, an examination showing 3400 bacteria per c.c. in the original certified milk and 1,400,000 in the milk in the thermos bottle after incubation over night. The occurrence of bucaal gonorrhœa in the new born is noted. Functional albuminuria is discussed briefly but very sensibly, especially in emphasizing the fact that when there is a pathology, the condition is no longer functional—

a truism, of course, but one not always appreciated. The "comforter" is denounced as pernicious. Dilatation of the colon is well described and illustrated. The importance of collecting urine from infants is emphasized and Chapin's device is described, though, unfortunately not very lucidly, as it is said to consist of a "circular opening ending in a funnel," etc. We trust that the next edition will tell us what there is around this hole and how it is attached to the patient. "Cancarem oris" is, of course, a misprint. This is the only error of the kind observed. The author's style is excellent and his work, though thorough and scientific, is highly practical.

---

Progressive Medicine, edited by Hobart A. Hare and Leighton F. Appleman, Philadelphia. Vol. 15, No. 3, Sept., 1913; Thorax, Wm. Ewart; Dermatology and Syphilis, Wm. S. Gottheil; Obstetrics, Edward P. Davis; Nervous System, Wm. G. Spiller. Published by Lea & Febiger, $6.00 per annum, four numbers.

This work, while based on current medical literature, is not merely an index or series of abstracts but a critical review of the progress of medical science. Obviously, the extent and importance of what may be termed discoveries, vary considerably from issue to issue and depend somewhat upon the field of the individual editors. Even in such departments as dermatology and obstetrics, in which it would appear that nothing of especial note had been accomplished within the last few months, one is surprised at the value, to the practitioner, of the reviews of clinical experience and collective studies.

---

Manual of Venereal Diseases. Introduction by Sir Alfred Keogh, History, Statistics, etc., by C. H. Melville, R. A. M. C. Clinical Pathology and Bacteriology by Sir Wm. Leischman; Clinical Course and Treatment by C. E. Pollock, R. A. M. C.; second edition, revised and largely re-written by L. W. Harrison, R. A. M. C. Published by Henry Frowde and Hodder & Stoughton, Oxford University Press, American Branch, 35 W. 32d St., N. Y. City; 318 pages, illustrated; $3.75.

While the authors are largely men of military experience, the work is equally applicable to ordinary civil practice. We note especially, descriptions of the demonstration of the spirochete, of the administration of salvarsan and of the treatment of gonorrhœa by electrically heated bougies. This is an excellent book, the only obstacle to its achieving wide popularity in this country, being the marked competition in this field.

HEADACHE, Its Varieties, Their Nature, Recognition and Treatment, by Dr. Siegmund Auerbach of Frankfurt, 3x a/M., translated by Ernest Playfair, M. B., M. R. C. P., published by Henry Frowde and Hodder & Stoughton, the Oxford University Press, American Branch, 35 W. 32d St., N. Y. City; 208 pages; $1.50.

This is a very useful little monograph, discussing the various forms of headache with special reference to etiology, which, of course, is the main point in determining a rational therapy. We are rather surprised not to find a definite reference to indol intoxication since, even if this substance is not the actual cause of headache, indol in the fæces and indican in the urine, frequently mark the occurrence of headache, especially in our personal experience, in cases that would otherwise be attributed to eye strain. We can scarcely agree that headache is especially characteristic of hyperchlorhydria and certainly not with the statement that this form of headache can be differentiated from migraine by the occurrence of the latter at intervals from childhood or adolescence. We do not understand that the term migraine indicates any particular pathogenic process, or that the headache occasionally noted in connection with hyperchlorhydria may not be a migraine. Hyperchlorhydria is very commonly observed in adolescence and has been reported even in infancy, though, sometimes, on rather inadequate evidence. These criticisms do not in any way detract from the value of the work. In fact, we rather prefer books that are more or less polemic and that naturally arouse a spirit of contradiction in the reader and thus make him think for himself.

---

PRACTICAL BACTERIOLOGY, BLOOD WORK AND ANIMAL PARASITOLOGY, by E. R. Stitt, A. B., Ph. G., M. D., Medical Inspector U. S. Navy. P. Blakiston's Son & Co., Philadelphia; third edition; four plates, 106 illustrations (513 separate figures); 410 pages; $1.50.

Even the fly leaves and inside of the covers are utilized for charts, but blank leaves are inserted for notes. The price of the book reperesents about one mill for each definite scientific fact presented. The author economizes words, but is generous with illustrations and ideas. He has the remarkable genius of presenting everything in its proper relations, but very briefly by the use of tables, and of following out associated lines of thought in the same way, or by terse notes. Like many of the rest of us, his motto seems to have been Completeness without Prolixity, but, unlike most of us, he has managed to live up to the motto.

Many writers give the impression of completeness until one attempts to follow their writing as a guide in practical work when it is discovered that the reader is brought up to the point at which he must consult some expert for some detail or further explanation. So far as we can judge from portions of this work in which we have had some experience, this book fulfills, as perfectly as any book can, the function of guiding the reader.

